# Rat-to-Elephant-to-Human Transmission of Cowpox Virus

**DOI:** 10.3201/eid1404.070817

**Published:** 2008-04

**Authors:** Andreas Kurth, Gudrun Wibbelt, Hans-Peter Gerber, Angelika Petschaelis, Georg Pauli, Andreas Nitsche

**Affiliations:** * Robert Koch Institute, Berlin, Germany; † Leibniz Institute for Zoo and Wildlife Research, Berlin, Germany;; ‡ Veterinär-und Lebensmittelüberwachung, Grimmen, Germany;; § Fachgebiet Gesundheitsamt des Landkreises Nordvorpommern, Grimmen, Germany

**Keywords:** Cowpox virus, zoonotic transmission, elephant, rat, human, natural reservoir, diagnostic, poxvirus, orthopoxvirus, elephantpox virus, letter

**To the Editor:** Despite the eradication of smallpox in the past century, other orthopoxviruses, such as monkeypox virus, vaccinia virus in Brazil, and cowpox virus (CPXV) in Europe (*1*), still infect humans. CPXV has been restricted to the Old World with wild rodents as its natural reservoir (*2*,*3*). Human CPXV infections are commonly described in relation to contact with diseased domestic cats, rarely directly from rats (*2*,*4*). Human infections usually remain localized and self-limiting but can become fatal in immunosuppressed patients (*5*). CPXV infections in captive exotic animals have been reported to be transmitted by rodents (*2*,*6*).

In February 2007, a circus elephant (*Elephas maximus*) in northern Germany exhibited disseminated ulcerative lesions of the skin and mucosal membranes (Figure, **panel A**) caused by CPXV infection; the elephant was euthanized after treatment attempts failed. Electron micrographs of negative-stained biopsy specimens of tongue lesions showed orthopoxvirus particles. The presence of orthopoxvirus after routine virus isolation in Hep2 cells was confirmed in direct immunofluorescence assay with orthopox-specific antibodies. The morphologic feature of hemorrhagic pocks on the chorioallantoic membrane (CAM) of infected embryonated hen’s eggs indicated CPXV. This finding was confirmed by sequence analysis of the complete hemagglutinin (HA) open reading frame (ORF), which showed 99% homology of 921 bp to CPXV isolated in 1984 from an elephant in Hamburg, Germany (Figure, **panel B**). A serum sample was drawn from the elephant 2 weeks before euthanasia. An indirect fluorescent antibody test (IFAT) detected immunoglobulin (Ig) G antibodies against the new corresponding elephant virus isolate (termed CPXV GuWi) with a titer of 1,260. According to the owner, the >40-year-old female elephant had never been vaccinated with vaccinia virus.

**Figure:**
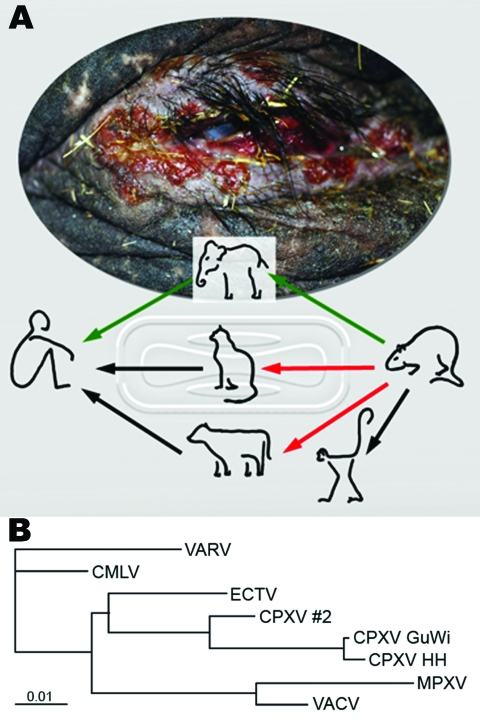
Route of cowpox virus (CPXV) transmission and phylogenetic analysis of orthopoxviruses. A) Disseminated ulcerative lesions of the skin around the eye of the circus elephant. Although transmission of CPXV has been confirmed from cats and cows to humans (black arrows) (*1*,*2*), transmission from rodents, commonly mice, to cats and cows is suspected but still unproven (red arrows) (*3*). Rats have been confirmed as vectors for CPXV transmission to monkeys and humans (*4*,*7*). A complete chain of CPXV infection is verified from rat to elephant and from elephant to human (green arrows). B) Phylogenetic tree of nucleotide sequences of the complete hemagglutinin open reading frame (921 bp) from CPXV isolates from the elephant and rat (CPXV GuWi), and additional poxviruses available in GenBank: VARV (variola major virus, strain Bangladesh-1975; L22579), CMLV (camelpox virus M-96, Kazakhstan; AF438165.1), ECTV (ectromelia virus, strain Moscow; AF012825.2), CPXV HH (cowpox virus cowHA68, Hamburg; AY902298.2), MPXV (monkeypox virus, strain Zaire-96-I-16; AF380138.1), and VACV, (vaccinia virus WR; AY243312). In addition, the complete sequence of the hemagglutinin gene obtained from a different human CPXV case (CPXV #2) found in that area is shown. Nucleotide sequences were aligned and analyzed by using the BioEdit software package (www.mbio.ncsu.edu/BioEdit/bioedit.htm). A multiple alignment was analyzed with the neighbor-joining method. The branch length is proportional to evolutionary distance (scale bar).

Eight days after the elephant’s death, a circumscribed lesion developed on the back of the right hand of a 19-year-old immunocompetent, healthy, unvaccinated animal keeper. CPXV was isolated from lesion fluid and was found to be homologous by using the HA ORF to CPXV GuWi. A convalescent-phase serum sample from the keeper taken 3 weeks later showed a significant increase in IgM (from 40 to 2,560), IgG (from 20 to 10,240), and neutralizing antibody (from <5 to 80) titers.

Further simultaneous investigations were undertaken to determine the source of infection. Because no felids were kept on the circus premises, the focus centered on wild rodents that had propagated and infested the area because of the mild winter. Six days after the elephant’s death, 4 rats (*Rattus norvegicus*) were caught and tested for orthopoxvirus antibodies. Although none of the rats had epidermal lesions or other pathologic changes indicative of a poxvirus infection, all were tested by IFAT and found to be serologically positive (IgG titers 40, 320, 2,560, and >10,240; IgM titers <5, <5, 160, and 2,560), indicating a recent infection in at least 2 animals. CPXV-typical pock morphologic features on the CAM could be visualized after homogenized liver and spleen of the animal with the highest titer was passaged 3 times. Infected CAM and original organ tissues (liver and spleen) showed CPXV by PCR and subsequent sequencing. The corresponding HA ORF displayed perfect homology to the viruses isolated from the elephant and the animal keeper.

We report CPXV infection in humans transmitted from an elephant, with rats as a probable source of the elephant’s infection (Figure, **panel A**). Although the animal keeper was infected by direct contact with the elephant, the exact transmission route from rat to elephant remains unclear. Nevertheless, rats have proven to be a natural reservoir for CPXV (*4*,*7*), and infections persisting for >3 weeks were shown for other rodents (*8*). No data about CPXV prevalence in rats are available, and no data for CPXV isolates from rats have been published in Germany. Therefore, further studies on rats as CPXV reservoir are needed to estimate the potential risk for infection among humans and exotic animals. Zoo and circus animals, especially elephants, seem to be highly susceptible to generalized CPXV infections. Although modified vaccinia virus Ankara was authorized in Germany to be used in vaccinating exotic animals (*9*), this case highlights the need for increased efforts toward general vaccination of potentially susceptible exotic animals in Europe.

The sequence identity of the HA ORFs also demonstrates a low mutation rate of CPXV after it crosses species barriers. As the Figure, **panel B**, infers, there is a phylogenetic difference between CPCV GuWi and CPXV from a human patient living in the same geographic area (CPXV #2), which indicates the cocirculation of >1 CPXV variant (*9*,*10*). Considering the extremely high virus load in infected animals, the broad host range of CPXV, and the abandoned vaccination against smallpox, this case emphasizes the risk among humans of acquiring CPXV infection (*6*). It also highlights the need for increased awareness regarding clinical features of orthopoxvirus infections and the importance of developing new antiviral drugs against orthopoxviruses.
